# Recent advances in PLLA-based biomaterial scaffolds for neural tissue engineering: Fabrication, modification, and applications

**DOI:** 10.3389/fbioe.2022.1011783

**Published:** 2022-11-01

**Authors:** Yuan Dai, Tingwei Lu, Minghao Shao, Feizhou Lyu

**Affiliations:** ^1^ Department of Orthopedics, Huashan Hospital, Fudan University, Shanghai, China; ^2^ Department of Oral and Craniomaxillofacial Surgery, Shanghai Ninth People’s Hospital, School of Medicine, Shanghai Jiao Tong University, Shanghai, China

**Keywords:** PLLA, Biomateriais, Scaffold, Neural tissue engineering, Peripheral nerve injury (PNI), spinal cord injury (SCI)

## Abstract

Repairing and regenerating injured neural tissue remains a worldwide challenge. Tissue engineering (TE) has been highlighted as a potential solution to provide functional substitutes for damaged organs or tissue. Among the biocompatible and biodegradable materials, poly-L-lactic-acid (PLLA) has been widely investigated in the TE field because of its tunable mechanical properties and tailorable surface functionalization. PLLA-based biomaterials can be engineered as scaffolds that mimic neural tissue extracellular matrix and modulate inflammatory responses. With technological advances, PLLA-based scaffolds can also have well-controlled three-dimensional sizes and structures to facilitate neurite extension. Furthermore, PLLA-based scaffolds have the potential to be used as drug-delivery carriers with controlled release. Moreover, owing to the good piezoelectric properties and capacity to carry conductive polymers, PLLA-based scaffolds can be combined with electrical stimulation to maintain stemness and promote axonal guidance. This mini-review summarizes and discusses the fabrication and modification techniques utilized in the PLLA-based biomaterial scaffolds for neural TE. Recent applications in peripheral nerve and spinal cord regeneration are also presented, and it is hoped that this will guide the future development of more effective and multifunctional PLLA-based nerve scaffolds.

## Introduction

Neural tissue injuries caused by trauma, accidents, and diseases have imposed an enormous psychological and economic burden on patients. The conventional therapies for central nervous system (brain and spinal cord) injuries have mainly focused on minimizing tissue and function loss, yet there is still no effective treatment to replace or regenerate the injured neural tissue (Katoh et al., 2019). The peripheral nervous system includes the rest of the nerves deriving from the spinal cord and the brain (Houshyar et al., 2019). Despite its intrinsic capability to repair itself, the functional recovery of peripheral nerves after injuries is often unsatisfactory (Yi et al., 2019).

Tissue engineering (TE) provides a promising strategy for developing functional substitutes for damaged or necrotic tissues (Hassanzadeh et al., 2018). Materials with excellent biocompatibility and biodegradability designed for TE are generally divided into natural and synthetic categories. Natural materials can be fabricated from extracellular matrix (ECM) proteins. ECM-based materials (e.g., collagen and laminin) have the capability to integrate well into injured tissues and facilitate axonal outgrowth (Haggerty et al., 2019; Yang et al., 2021). Natural materials also include non-ECM substances like chitosan whose cationic nature makes electrostatic reactions with proteoglycans and glycosaminoglycans in the body (Ehterami et al., 2021). Natural materials have been utilized for various applications of neural TE including peripheral nerve injury (PNI) and spinal cord injury (SCI). However, natural materials tend to degrade rapidly *in vivo* and have relatively low mechanical strength (Kaplan and Levenberg, 2022). Synthetic materials include poly-L-lactic-acid (PLLA), polycaprolactone (PCL), polyethylene glycol, and many others. Their properties can be modified more easily than natural materials. For instance, porosity, rigidity, and degradation rate can be altered to match demands of different tissues. However, synthetic materials may require surface functionalization as they are generally lack of integrin-binding molecules (Führmann et al., 2017). Among the synthetic materials, PLLA is a Food and Drug Administration (FDA)-approved medical material and has been extensively studied because of its low cost, ease of fabrication, tunable mechanical properties, and tailorable surface functionalization (Lin et al., 2018; Capuana et al., 2022). Additionally, PLLA can be easily blended with other materials or bioactive polymers to optimize its surface bioactivity (Jahromi et al., 2020; Siriwardane et al., 2021). Furthermore, PLLA-based materials can be fabricated into two-dimensional or three-dimensional (3D) scaffolds with stable nanofibrous and porous topology which provide the biomimetic structures for nerve cell adhesion and growth while supporting neural tissue formation (Almansoori et al., 2020; Behtaj et al., 2022; Weir et al., 2022). Moreover, PLLA-based scaffolds have the potential to be combined with various therapeutic approaches such as drug delivery, electrical stimulation, magnetic stimulation, and stem cell therapy, which have been explored in nerve repair and regeneration (Zhang et al., 2017; Chen et al., 2020; Jedari et al., 2020; Rahimi-Sherbaf et al., 2020; Tonellato et al., 2020).

**FIGURE 1:**
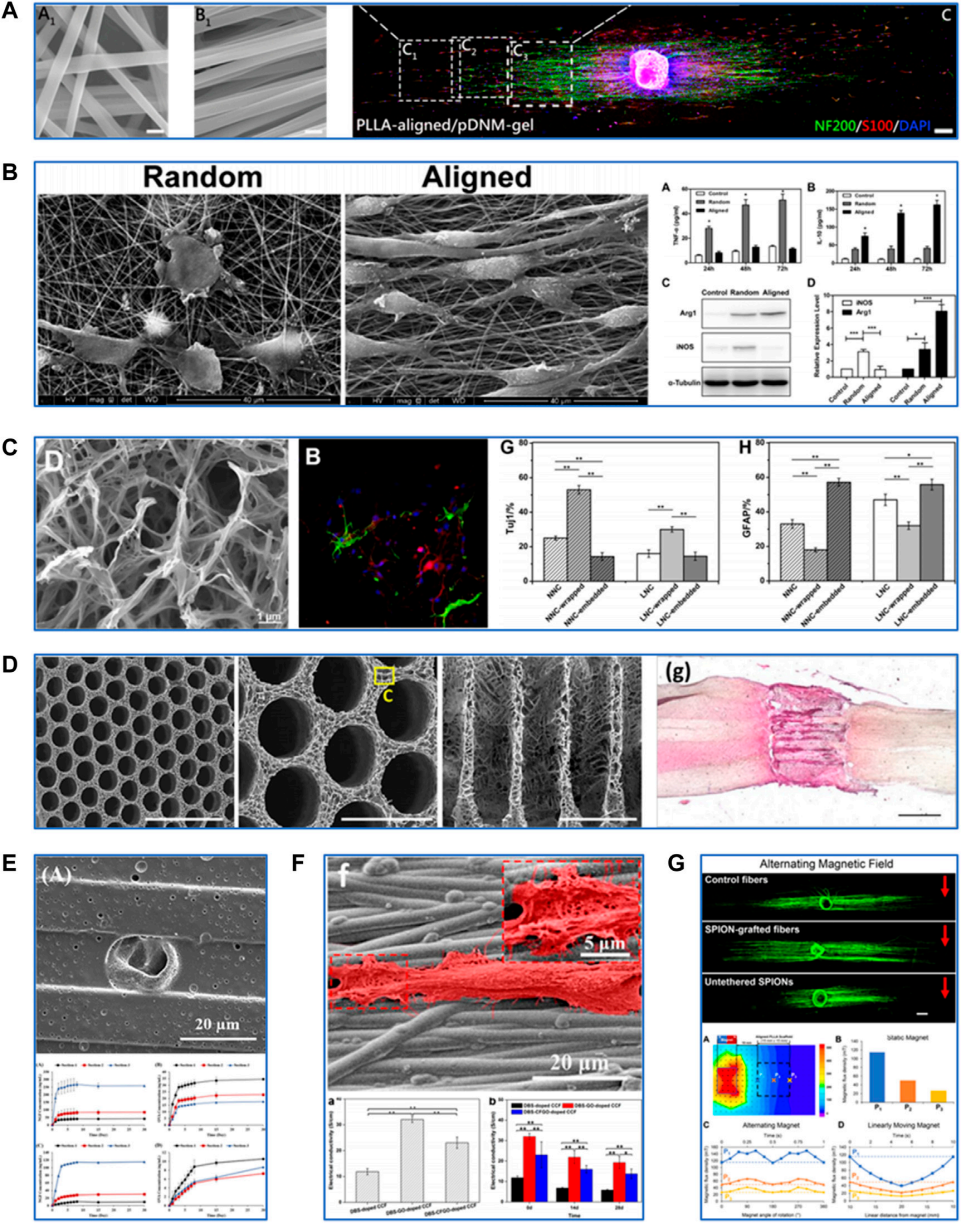
Fabrication and modification techniques of PLLA-based scaffolds for neural tissue engineering. **(A)** Characterization of PLLA-based scaffolds fabricated by electrospinning and their effects on neurite growth and Schwann cell migration. Reproduced with permission from Ref. (Chen S. et al., 2019). Copyright 2019, American Chemical Society. **(B)** Characterization of PLLA-based scaffolds fabricated by electrospinning and their effects on macrophage polarization. Reproduced with permission from Ref. (Jia et al., 2019). Copyright 2018, Elsevier. **(C)** Characterization of PLLA-based scaffolds fabricated by thermally induced phase separation and their effects on differentiation of neural stem cells into neurons. Reproduced with permission from Ref. (Liu et al., 2018). Copyright 2017, Elsevier. **(D)** Characterization of PLLA-based scaffolds fabricated by additive manufacturing and their effects on spinal cord regeneration. Reproduced with permission from Ref. (Kaplan et al., 2020). Copyright 2020, Elsevier. **(E)** Modification of PLLA-based scaffolds by drug delivery. Reproduced with permission from Ref. (Uz et al., 2017). Copyright 2016, Elsevier. **(F)** Modification of PLLA-based scaffolds by electrical stimulation. Reproduced with permission from Ref. (Shang et al., 2019). Copyright 2019, American Chemical Society. **(G)** Modification of PLLA-based scaffolds by magnetic stimulation. Reproduced with permission from Ref. (Funnell et al., 2021). Copyright 2021, Elsevier.

**FIGURE 2:**
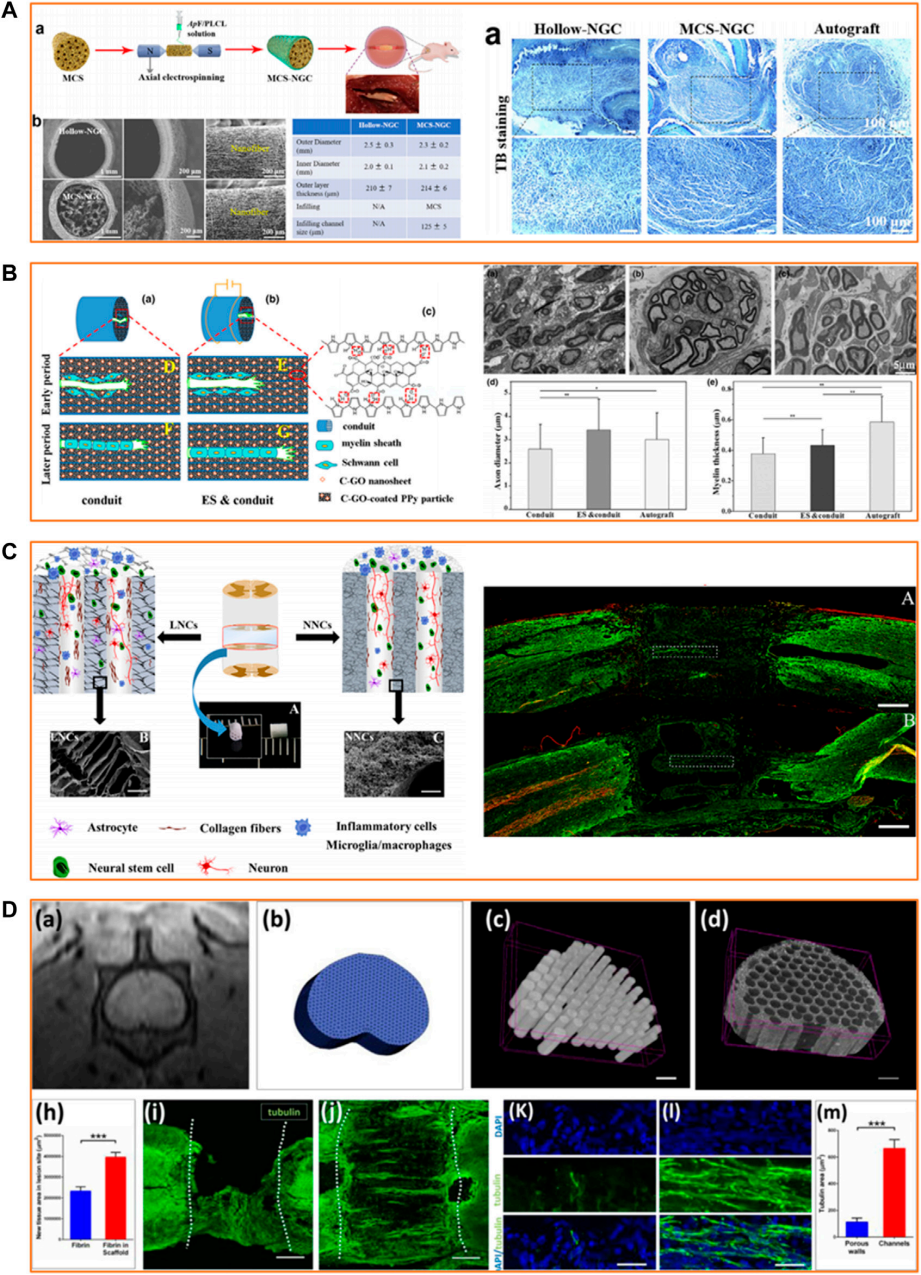
Applications of PLLA-based scaffolds in peripheral nerve and spinal cord regeneration. **(A)** Effective guiding support provided by biomimetic PLLA-based scaffolds for peripheral nerve regeneration. Reproduced with permission from Ref. (Wang et al., 2020). Copyright 2020, Elsevier. **(B)** Satisfying neurite growth induced by CGO/Ppy/PLLA scaffold under electrical stimulation for peripheral nerve regeneration. Reproduced with permission from Ref. (Chen X. et al., 2019). Copyright 2019, John Wiley and Sons. **(C)** Recruitment of endogenous stem cells by PLLA-based multi-channel scaffolds for spinal cord regeneration. Reproduced with permission from Ref. (Sun et al., 2019b). Copyright 2018, Elsevier. **(D)** Facilitation of axonal growth by a combination of PLLA-based scaffolds and stem cell therapy for spinal cord regeneration. Reproduced with permission from Ref. (Kaplan et al., 2020). Copyright 2020, Elsevier.

There have been increasing attempts to apply PLLA-based scaffolds in the neural TE field, but a comprehensive review of this subject does not exist. This mini-review summarized and discussed the techniques used to fabricate and modify PLLA-based scaffolds for neural TE (Figure 1). It also presented recent applications of PLLA-based scaffolds in treating peripheral nerve injury (PNI) and spinal cord injury (SCI) (Figure 2). Lastly, a brief outlook for improvement was suggested for future studies.

## Techniques used in the fabrication of PLLA-based scaffolds for neural tissue engineering

### Electrospinning

The electrospinning process involves the application of high voltage power between the spinneret of the polymer solution-loaded syringe and the receiving device, drawing continuous nanofibers out of the spinneret. It was reported that the electrospun PLLA/gelatin scaffold could promote the proliferation and glial cell-derived neurotrophic factor (GDNF) secretion of Schwann cells (SCs), which is essential for the survival of damaged neurons and regeneration of axons in PNI (Niu et al., 2021). Cell behaviors are strongly regulated by surface topographic features and nanoscaled cues of the scaffolds. Aligned electrospun fibers and similar microstripes might contribute to the guided cell adhesion and migration process. Cells could align predominantly along nanoscale grooves and ridges, whereas with an equally probability in all directions on a smooth surface (Yao et al., 2013). Yao et al. examined the effects of width and arc radius of the microstripes on guided cell migration (Yao and Ding, 2020). Their results showed that straight microstripes with 20-μm width could enhance migration while wider or wavy microstripes with a smaller radius caused multiple-row cell arrangement and narrower microstripes restricted adhesion of cells, thus relatively reducing migration. Aligned electrospun PLLA fibers could increase the alignment degree of neurite outgrowth and neurite length of PC12 cells (Xuan et al., 2021). Aligned electrospun PLLA fibers coated by decellularized peripheral nerve matrix gel facilitated further SC adhesion and migration and faster neurite extension compared with the random nanofibrous structures (Chen S. et al., 2019). Jia et al. (2019) have proposed a novel mechanism by which aligned PLLA/polycaprolactone (PCL) nanofibers modulate macrophage phenotypes by inducing the development of the pro-healing type. The macrophages polarized by the aligned PLLA/PCL nanofibers also promoted SC proliferation and migration *in vitro*. The PLLA-based electrospun nanofibrous scaffolds, especially those with aligned nanofiber arrangement, can promote nerve cell proliferation, guide neurite extension, and modulate macrophage polarization towards anti-inflammation.

### Thermally induced phase separation

Thermally induced phase separation (TIPS) is a process of thermodynamic dissociation of a homogeneous polymer-solvent system to the polymer-rich and solvent-rich phases by either liquid-liquid phase separation (LLPS) or solid-liquid (SLPS) phase separation (Zeinali et al., 2021). TIPS allows one to obtain scaffolds with high porosity, which is essential for transporting the cell metabolites and nutrients. While LLPS procedures led to the formation of nanofibrous microstructures in the PLLA scaffolds, SLPS procedures resulted in the formation of ladder-like microstructures (Zeng et al., 2014). Liu et al. used gelatin to wrap or embed the PLLA scaffolds of two microstructures by chemical crosslinking with genipin (Liu et al., 2018). The results showed that the PLLA scaffold of nanofibrous microstructure wrapped with gelatin maintained its micromorphology and had improved flexibility and a more reasonable degradation rate. Moreover, neural stem cells (NSCs) expressed a higher level of differentiation into neurons on it. Another study utilized the LLPS method to fabricate a highly microporous and nanofibrous PLLA/chitosan scaffold (Ehterami et al., 2021). Compared with the pure PLLA, the PLLA/chitosan composite scaffold demonstrated increased adhesion and proliferation and decreased free radical generation of human neuroblastoma cells. These findings suggest TIPS provide PLLA-based scaffolds with appropriate microstructures to promote nerve cell growth and maintain their functions.

### Additive manufacturing

Additive manufacturing (AM), also known as 3D printing or rapid prototyping, fabricates materials from a digital model, usually by a layer-upon-layer addition of materials. AM-based fabrication allows for constructing a scaffold with a well-controlled size and structure. A previous study prepared a novel PLLA scaffold incorporated with Polypyrrole (Ppy) nanoparticles by extrusion-based low-temperature deposition 3D printing (Ma et al., 2019). The composited scaffold showed optimized structure and good biocompatibility and maintained excellent electrical conductivity, which has the potential for axonal growth and guidance. Furthermore, AM-based fabrication also meets the dem and for personalized treatment. Kaplan et al. (2020) synthesized a highly ordered PLLA/poly (lactic-co-glycolic acid) multichannel scaffold using a 3D printed sacrificial construct based on the magnetic resonance imaging of the rat coronal spinal cord segment. This anatomically personalized scaffold fitted accurately into the spinal cord lesion *in vivo* and supported the growth and guidance of axons derived from induced pluripotent stem cells (iPSCs). AM offers a promising way to build a PLLA-based scaffold with enhanced precision and control over architecture.

## Techniques used in the modification of PLLA-based scaffolds for neural tissue engineering

### Drug delivery and controlled release

PLLA has been widely used in controlled drug release because it has a relatively slow biodegradation process and can be fabricated into a scaffold with high porosity and surface-to-volume ratio. Nerve growth factor (NGF) is the most common growth factor applied in nerve cell proliferation and neural tissue repair. Uz et al. developed a porous PLLA scaffold film with longitudinal micropatterns, which possessed both an NGF gradient on the film surface and NGF-loaded polyanhydride microparticles with controlled release properties (Uz et al., 2017). The release kinetic studies proved the ability of this strategy to enhance sustained NGF release. According to Uz et al. (2017), PC12 cells showed aligned and guided neurite extension parallel to the direction of the micropattern and the NGF gradient. Angiogenesis also plays a pivotal role in nerve tissue repair and regeneration. Xia et al. prepared a core-shell fibrous scaffold using the emulsion electrospinning technique, and they introduced NGF into the core of the scaffold to achieve NGF release in a controlled manner (Xia and Lv, 2018). Then the scaffold was functionalized with vascular endothelial growth factor, which can stimulate angiogenesis and SC proliferation. This dual-delivery scaffold promoted the differentiation of iPSC-derived neural crest stem cells *in vitro* and further improved neovascularization and nerve healing in rats with a 10-mm sciatic nerve defect 3 months post-implantation. Anti-inflammatory drugs have also been extensively used, and this is because the inflammatory response is another therapeutic target for neural tissue injury. Bighinati et al. constructed an injectable dual-drug delivery PLLA scaffold. Ibu and T3 were blended in polymeric solutions, then co-electrospun on the same collector (Bighinati et al., 2020). This scaffold released 48 μg/ml of Ibu over 14 days, with an estimated daily release of 3.4 μg/ml and 50 ng/ml of T3 every day with an estimated release of 3.5 ng/ml. The other results revealed that this dual-drug delivery scaffold decreased glutamate release and glial scar formation while improving locomotion recovery in a rat model of spinal cord contusion injury 7 weeks post-implantation. These studies indicate that PLLA-based scaffolds are excellent candidate materials for growth factors and drug delivery to treat neurological injuries.

### Electrical stimulation

The application of electrical stimulation (ES) has been extensively investigated in nerve regeneration (Gopalakrishnan-Prema et al., 2020). Piezoelectric materials and conductive polymers have been tested to provide extra electrical signals in TE. PLLA itself possesses suitable piezoelectric properties. Barroca et al. (2018) utilized an electrical poling device to develop aligned electrospun PLLA nanofibers with the negatively or positively charged surface. From their findings, polarization quantification revealed values up to 60 μC/m^2^ and stability for up to 6 months for these polarized nanofibers. Furthermore, negatively or positively polarized PLLA nanofibers increased the proliferation and differentiation of neuroblastoma SH-SY5Y cells compared with non-polarized nanofibers. Another study has proposed an NSC culture system in which electrons are released to the surface of PLLA nanofibers driven by ultrasonic irradiation, their results demonstrated a clear piezoresponse in fibers, and the local effective piezoelectric signals reached 4.2 mV (Lu et al., 2022). In this culture system, NSCs could maintain expansion and stemness without growth factors, which provides a novel method for *in vitro* expansion of NSCs with inexpensive materials. Ppy is a common conductive polymer applied in the TE scaffold. Sun et al. (2019a) prepared a hollow tubular scaffold made of electrospun PLLA/PCL/silk fibroin coated with Ppy. The histological results showed that this Ppy-coated scaffold could promote SC proliferation and myelin sheath formation after 4 weeks of being implanted into the gap of the sciatic nerve defect in rats. However, some studies suggested that the electrical conductivity of Ppy was unsustainable and not strong enough. Thus, some researchers introduced graphene oxide (GO) and its derivatives into a Ppy-coated PLLA scaffold to maintain and enhance electrical conductivity (Shang et al., 2019). Li et al. (2020) synthesized a carboxylic GO (CGO)/Ppy/PLLA electrospun scaffold film. Their results showed increased proliferation and alignment of SCs on this film compared with Ppy/PLLA film. These findings suggest that the PLLA-based scaffold has excellent potential as a piezoelectric material or a conductive polymer carrier for neural TE.

### Magnetic stimulation

Several studies have suggested that magnetic stimulation could accelerate neurite outgrowth and promote neurogenesis, and the mechanism is thought to be associated with charged particles or molecules in or around the cells (Semeano et al., 2022). Superparamagnetic iron oxide nanoparticles (SPIONs) have been widely explored in the biomedical fields resulting in their reliable biocompatibility and remarkable superparamagnetic properties (which need an external magnetic field for activation). Johnson et al. (2019) successfully incorporated SPIONs into aligned electrospun PLLA nanofibers. The results showed that nanofibers with SPIONs at 6% of the PLLA weight had shorter reorientation times and higher alignment degrees in response to the static magnetic field. Then, they cultured dorsal root ganglia (DRG) and SPION nanofibers together within a 3D hydrogel system in a 0.2T static magnetic field. DRG demonstrated improved orientation and length of neurites with SPION nanofibers compared with SPIONs or nanofibers alone. Funnel et al. further proved that applying an alternating magnetic field increased neurite length on aligned SPION-grafted PLLA nanofibers (Funnell et al., 2021). Therefore, magnetic stimulation provides new insights into the modulation of PLLA-based scaffold for neural TE.

## Applications of PLLA-based scaffolds in peripheral nerve and spinal cord regeneration

### Applications of PLLA-based scaffolds in peripheral nerve regeneration

PNI results in the loss of sensory or motor functions, adding severe physical, psychological and economic burden on patients (Lee et al., 2022). Nerve autografts remain the clinical gold standard for treating PNI; however, nerve autografts have significant limitations such as donor site morbidity, limited donor supply, and mismatch between the donor and the recipient site (Zhang et al., 2020a). Scaffolds with PLLA as the primary polymer effectively bridge the gap in PNI. A hollow tubular CGO/Ppy/PLLA scaffold fabricated by electrochemical deposition showed stable electrical conductivity and tensile strength 4 weeks post-immersion (Chen X. et al., 2019). The *in vivo* experiment results indicated that the scaffold under ES could promote the migration and extension of SCs, stimulate myelin sheath formation, and induce muscle reinnervation and neurite regeneration in the sciatic nerve defect model of rats. Researchers have also been making efforts toward complex structure design to mimic the morphology of native peripheral nerves. Antheraea pernyi silk fibroin (ASF) is a biodegradable and biocompatible natural macromolecule containing arginine–glycine–aspartic acid tripeptide sequence which is reported to own good cell adhesion ability (Yao et al., 2022). Zou et al. (2021) utilized regenerated ASF (RASF) solution to fabricate electrospun fibrous scaffolds which was demonstrated to favor proliferation, permeation and migration of SCs . Wang et al. (2020) fabricated a biomimetic scaffold with parallel microchannels (φ = 125 μm) from fragmented nanofibers of RASF/PLLA/PCL in combination with the nanomaterial graphene oxide. Their results showed that this composite scaffold could promote SC proliferation and migration *in vitro* and lead to the formation of myelinated nerves and microvessels similar to autografts in the rat model. Despite the intrinsic and extrinsic mechanisms that allow peripheral nerves to heal spontaneously, the regeneration capacity of peripheral nerves is generally limited (Kaplan and Levenberg, 2022). Hence, harnessing stem cells for regenerating an injured nerve has been studied (Dębski et al., 2021). Zhang et al. constructed a soy protein isolate/PLLA nanofibrous scaffold and seeded bone marrow stem cells (BMSCs) overexpressing brain-derived neurotrophic factor and BMSCs overexpressing GDNF in the inner wall of the scaffold (Zhang et al., 2020b). Histological and electrophysiological evaluation 3 months after scaffold implantation showed significant improvement in the sciatic nerve repair in the rat, indicating that the PLLA-based scaffold has the potential to be combined with stem cells to treat PNI.

### Applications of PLLA-based scaffolds in spinal cord regeneration

SCI is one of the most severe neurological injuries that cause temporary or even permanent loss of sensation and movement below the injured segment. SCI treatment demands a combinatorial approach for neuroprotection and neuroregeneration (Abbas et al., 2020). PLLA-based scaffolds have been demonstrated to be a promising candidate for spinal cord repair. Sun et al. (2019b) devised multichannel PLLA scaffolds with ladder-like or nanofibrous channel walls as a regenerative therapeutic strategy for SCI. Their findings revealed that PLLA scaffolds of micro/nano-architectures significantly alleviated the infiltration of macrophages and decreased the accumulation of glial scars. This is in addition to recruiting the endogenous stem cells and facilitating the axonal growth in the complete spinal cord transected injury rat model. The anti-inflammatory effects of the PLLA-based scaffold can further be enhanced by blending it with chemical compounds or drugs to maximize the neuroprotective properties (Dolci et al., 2021; Xiao et al., 2021). Unlike the peripheral nerve, the adult spinal cord has minimal regeneration capacity (Kaplan and Levenberg, 2022). Hence, stem cells, in conjunction with scaffolds, have been widely investigated as a neuroregenerative method for the repair of SCI. In a study by Miri et al., an electrospun PLLA scaffold was tested using NSC proliferation and differentiation *in vitro* as outputs (Miri et al., 2021). The results proved that a nanofibrous PLLA scaffold could support the normal growth, proliferation, and differentiation of NSCs. In another study, Moazamiyanfar et al. demonstrated that a PLLA scaffold covered with platelet-rich plasma showed low toxicity toward iPSCs and induced high expression of gene markers of neuronal cells in the differentiated iPSCs (Moazamiyanfar et al., 2022). Furthermore, a PLLA-based scaffold loaded with stem cells could promote the restoration of neural network and gait function in the rat model of SCI (Kaplan et al., 2020). A mixture of scaffolds based on PLLA and stem cell therapy provides a promising strategy for SCI repair.

## Conclusion and perspectives

The characteristics of PLLA-based materials include biocompatibility, biodegradability, tunable microstructures, and adjustable surface properties, which gives them advantages in the neural TE field. PLLA-based scaffolds can be fabricated into nanofibers to provide a support structure and an attachment site for nerve cells (Gao et al., 2013; Qiang et al., 2021). The porous PLLA-based scaffolds produced by TIPS present good interconnectivity and cell permeability and provide a stable platform for drug delivery (Salehi et al., 2019). With the aid of AM, biomimetic PLLA-based scaffolds with 3D structure better adapt to the defect in neurological injuries. Besides fabrication techniques, various modification methods can be adapted to enhance the bioactivity of PLLA-based scaffolds to satisfy the complex microenvironmental needs of neural tissue regeneration. Specifically, we reviewed PLLA-based scaffolds modified for drug delivery, electrical stimulation, and magnetic stimulation. The PLLA-based scaffolds can repair the PNI and restore the movement function in the rat sciatic nerve defect model. Meanwhile, with the ability to recruit endogenous NSCs, maintain the stemness of exogenous NSCs, and facilitate the differentiation of iPSCs into the neural lineage, the PLLA-based scaffolds also have the potential to repair the injured spinal cord in the rat.

Neural tissue repair and regeneration is a highly complex process, and the deeper biochemical and biophysiological mechanisms behind it need to be investigated to design PLLA-based scaffolds with more advanced properties. Researchers have attempted to combine gene therapy, a burgeoning approach to neurological diseases, with a PLLA-based scaffold to enhance nerve regeneration at the molecular level (Wang et al., 2022). Sufficient vascularization also contributes to neuroregeneration. PLLA composite scaffolds have been widely applied in artificial blood vessels, and there are emerging studies to fabricate prevascularized PLLA-based scaffolds for angiogenesis-mediated nerve regeneration (Shen et al., 2021; Song et al., 2021). Moreover, future studies need to look into developing novel PLLA-based composites blended with other bioactive synthetic/natural polymers and combining various modification strategies corresponding to the strict demands for guiding nerve cell migration and axonal extension. Nevertheless, with the rapid advancement in fabrication techniques, PLLA-based scaffolds will be more adaptable for a broader range of applications. Finally, the clinical effects of the PLLA-based scaffold for neural TE should be assessed in larger mammals to better underst and the dynamic changes during the repair and regeneration process.
